# miRNAs in breast cancer tumorigenesis (Review)

**DOI:** 10.3892/or.2011.1611

**Published:** 2011-12-23

**Authors:** ZHONG JU ZHANG, SHI LIANG MA

**Affiliations:** College of Biological Science and Biotechnology, Shenyang Agricultural University, Shenyang 110866, P.R. China

**Keywords:** miRNA, breast cancer

## Abstract

miRNAs are small, endogenous, non-coding RNAs that negatively regulate protein-coding mRNAs at the post-transcriptional level. It is estimated that in humans thousands of miRNAs are expressed and more than 700 miRNAs have been described to date. About 50% of annotated human miRNAs are detected in regions of fragile sites, which are associated with cancer. The available evidence has shown that miRNAs widely participate in the development or progression of many types of cancers, including breast cancer. The role of miRNAs in breast cancer has been widely investigated; here, we will focus on what is known about the working mechanism of miRNAs in different stages of breast cancer development.

## 1. Introduction

miRNA was initially discovered as a small temporal RNA (stRNA) in *C.elegans* in 1993 (1). However, not much attention was paid to this finding until seven years later when Let-7 (the second miRNA) was identified (2). In the following years, researchers became aware that miRNAs were a large family of small non-coding RNAs that exist in species ranging from plants to humans (3–8). Correspondingly, the functions of miRNAs were found to not be limited to temporal regulation, but were shown to be implicated in various biological processes, including cell cycle (9,10), proliferation (11), apoptosis (12,13) and development (6). In 2002, Calin and colleagues (14) reported the first direct evidence of miRNA playing a role in human cancer; they found that miR-15 and miR-16 contribute to chronic lymphocytic leukemia. Subsequently, more examples of miRNA correlated with human cancers were noted. Iorio and colleagues (15) first demonstrated miRNA dysregulation in human breast cancer by miRNA microarray; they found that miR-10b, miR-125b, and miR-145 were down-regulated, while miR-21 and miR-155 were up-regulated, suggesting that these miRNAs may act as potential tumor suppressor genes or oncogenes, respectively. Following this finding, more functional studies had identified specific miRNAs as pivotal regulators in different stages of breast cancer development (initiation, progression and metastasis). In this review we will summarize the current understanding regarding the functions of miRNAs in breast cancer tumorigenesis.

Finally, based on these experimentally validated specific breast cancer-associated miRNAs and their gene targets, we summarize stage-specific miRNA functions of breast-derived cells and tissues in different phases (Table I). This approach reveals that some miRNAs, such as miR-21, play a key role in all phases of breast cancer tumorigenesis. Other miRNAs, such as miR-30, miR-17-5p, miR-9, are phase-specific. It suggests that regulation of miRNAs themselves at specific stages may be crucial for breast cancer tumorigenesis.

## 2. microRNAs as regulators in breast cancer initiation

**Figure 1:**
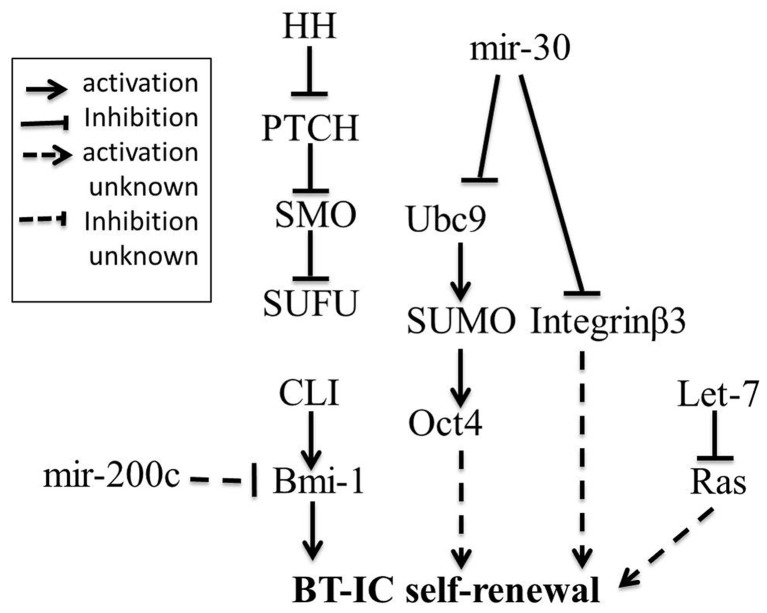
A simple model for regulation of breast cancer initiating cells (BT-IC) self-renewal in breast cancer. The Hedgehog (HH) pathway regulates the self-renewal of BT-IC via the downstream targets: Patched (PTCH), Smoothened (SMO), Suppressor of Fused (SUFU), CLI and Bmi-1. miR-200c is also associated with this pathway. miR-30 and Let-7 may modulate BT-IC self-renewal by the pathway shown here.

Currently, it is universally acknowledged that cancers may arise from cancer stem cells, also termed as tumor-initiating cells (T-IC), which are the primary cellular components within a tumor that drives disease progression and are characterized by their stem-like ability to self-renew (16). Evidence of breast cancer-initiating cells (BT-IC) was reported by Al-Hajj and Clarke (17). Such cells may be responsible for breast cancer initiation. However, how the self-renewal of BT-ICs is regulated remains obscure. A previous study showed that the regulation of the self-renewal of breast cancer stem cells is associated with the Hedgehog pathway. Bmi-1 is a downstream target of Hedgehog pathway. Furthermore, Bmi-1 has been shown to be required for self-renewal (18,19). These findings clearly indicate that the Hedgehog pathway activates breast cancer stem cell self-renewal by Bmi-1. A recent study has implicated several miRNAs in the regulation of BT-IC self-renewal. These miRNAs include miR-200c, Let-7, miR-30, but presently, little is known about the mechanism by which it functions to regulate BT-IC self-renewal. What is clear is that miR-200c strongly suppressed the ability for self-renewal of breast cancer stem cells (20). Studies also show that lack of Let-7 is required for self-renewal in breast cancer stem cells; moreover, Ras is determined as the direct target of Let-7 and its silencing contributes to loss of BT-IC self-renewal (21). More recently, it is demonstrated that up-regulated expression of miR-30 in breast cancer-initiating cells inhibits their self-renewal capacity by reducing the ubiquitin-conjugating enzyme 9 (Ubc9). Ubc9 has been shown to be specific for small ubiquitin-related modifier (SUMO) activation. SUMO may up-regulate Oct4 by stabilizing its structure (22,23). Oct4 overexpression induced by Ubc9 contributes to the self-renewal (24). Integrin β3 (ITGB3) is another direct target of miR-30, which contributes to apoptosis (22). A previous report has shown that unligated ITGB3 recruits caspase-8 to the cell membrane and activated caspase-8-mediates apoptosis in a death receptor-independent manner (25), while miR-30 induces apoptosis not in the death receptor-independent manner but through an unclear pathway. In addition, a recent study indicates that integrins play a role via directly regulating the ability of BT-ICs to self-renew during the initial steps of breast cancer tumorigenesis (26). These findings support two greatly simplified models of regulation of BT-IC self-renewal (Fig. 1). The identification of miRNAs functioning as regulators of BT-IC self-renewal partially expand our understanding of the regulation of breast cancer initiation.

Although the molecular mechanisms by which miRNAs play a crucial role in tumor progression and metastasis have been studied in great detail over the last decades, the role of miRNAs in the early events of tumorigenesis has only recently been demonstrated. As tumor formation is a multi-step process, the initiating events may facilitate the development of effective targeted therapeutic strategies for cancer.

## 3. Roles of microRNAs in breast cancer progression

Cancer stem cells drive tumor progression and heterogeneity by proliferating and generating some differentiated cancer cells (27). These differentiated cancer cells will gain the ability to anti-apoptosis and full out of the control of the normal cell cycle during cancer progression. Here, we will highlight those miRNAs identified as regulators of anti-apoptosis and of the cell cycle in breast cancer progression.

### Anti-apoptosis

**Figure 2:**
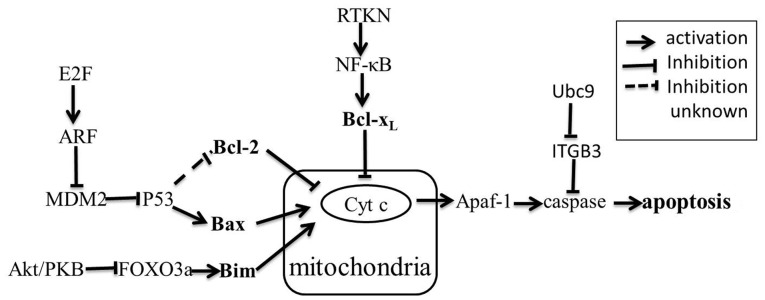
Regulation of apoptosis by several pathways. When pro-apoptotic members of the Bcl-2 family, such as Bax and Bak, are activated by some pathway, mitochondria release cytochrome c. Cytochrome c in turn associates with Apaf-1 and then caspase to trigger apoptossis. Anti-apoptotic members, such as Bcl-2 and Bcl-x_L_, will keep cytochrome c in the mitochondria inhibiting the apoptotic pathway. The Ubc9/ITGB3 pathway activates apoptossis in a mitochondria-independent manner.

In normal breast tissue, apoptosis plays a key role for performing the normal functions. The mechanism of apoptosis still needs to be fully investigated. There is evidence that mitochondria play an essential role in the apoptotic process (Fig. 2). Several pathways contribute to apoptosis, but the best characterized are the Akt/PKB pathway, RTKN/NF-κB survival pathway and the p53-mediated apoptosis pathway (28–30). Emerging research shows that miRNAs are involved in these pathways. Bcl-2 family proteins can be thought of as the central factors of the apoptotic pathway. The Bcl-2 family is comprised of many proteins, which can be classified into three functional groups. Group I members, including Bcl-2 and Bcl-x_L_, possess anti-apoptotic activity; group II members, including Bax and Bak, are characterized by pro-apoptotic activity; group III members, such as Bim and Bad, also possess pro-apoptotic activity (31). After the identification of the down-regulation of miR-15/16 promoting anti-apoptosis via up-regulating Bcl-2 expression in leukemias and lymphomas, more miRNAs promoting anti-apoptosis by directly or indirectly regulating Bcl-2 family proteins have been observed in many types of cancer, including breast cancer (32).

**Figure 3:**
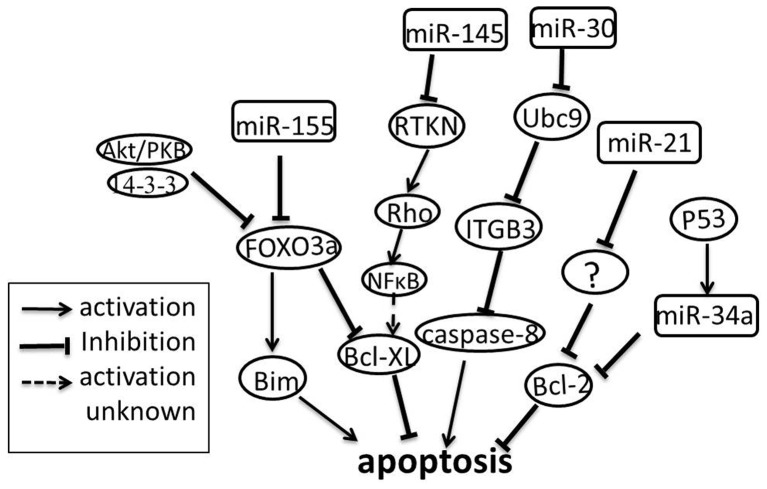
Model for microRNA-involved apoptosis in breast cancer. miR-155, miR-145, miR-21 and miR-34a regulate apoptosis in a mitochondria-dependent manner, but miR-30 does not.

Studies by Si and colleagues (33) showed that treatment of MCF-7 breast cancer cells with anti-miR-21 causes cell apoptosis. Moreover, they detected a lower level of Bcl-2 expression both at the mRNA and at the protein level in the anti-miR-21-transfected MCF-7 cells as well as in tumors derived from the MCF-7 cells transfected with anti-miR-21 (33). These investigations reveal that miR-21 may act as an anti-apoptotic factor by indirectly targeting Bcl-2, consistent with the previous report for glioblastoma cells (34). In contrast, miR-145 was down-regulated in MCF-7 cells, and overexpression of miR-145 suppressed MCF-7 cell growth and induced apoptosis (35). RTKN was confirmed as a direct target of miR-145 in MCF-7 cells (35). RTKN further enhanced the expression of Bcl-x_L_ by activating NF-κB (29). In normal cells, miR-145 suppresses RTKN to maintain its low expression level; while in cancer cells, miR-145 is down-regulated, leading to increased cell survival. As mentioned above, miR-21 is up-regulated and miR-145 is down-regulated in breast cancer cells, but each contributes to anti-apoptosis via different pathways. How the two miRNAs cooperate with each other to promote apoptosis resistance is fully unknown, maybe the identification of the direct target of miR-21 in regulating Bcl-2 expression will shed a new light on our understanding of the complex regulatory mechanism. Similarly, miR-155 is also up-regulated in breast cancer cells, and also contributes to anti-apoptosis. Kong *et al* (36) demonstrated that miR-155 induces cell survival by targeting FOXO3a in breast cancer. FOXO3a is a major member of the forkhead transcription factor family characterized by a distinctive forkhead DNA binding domain. They are localized in the nucleus without growth factor stimulation, and function as transcription factors to enhance apoptosis by promoting the expression of Bim (pro-apoptotic member of the Bcl-2 family). When phosphorylated by protein kinase B, FOXO3a is assembled into a complex with the 14-3-3 protein. The complex is then exported from the nucleus and loses its pro-apoptotic function (36,37). miR-34a is shown to play a role in the p53-mediated apoptosis of human lung cancer cells. Further study suggests that p53 can directly regulate the gene encoding miR-34a. Bcl-2 is a target of miR-34a and loss of miR-34a protect cells from apoptosis (Fig. 3) (30). miRNAs involved in p53-mediated apoptosis are also validated in other cancer cell lines (38). However, little is known about such miRNAs in breast cancer. A recent study examined whether miR-34a is necessary to induce apoptotic cell death in a breast cancer cell line. This process seems to be associated with p53, but the mechanism remains largely unknown (39). Cleary, the events of miRNA-mediated anti-apoptosis in breast cancer is still not yet fully realized and many advances should be anticipated in this field in the foreseeable future.

### Cell cycle dysregulation

**Figure 4:**
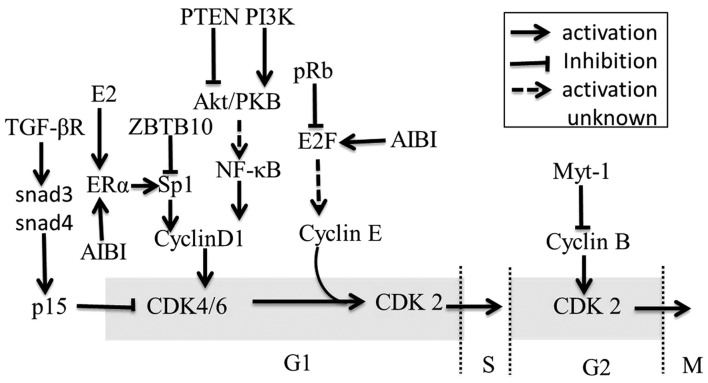
Regulation of the cell cycle. Cyclins play a key role in different pathways.

Cancer cells are characterized by deregulated cell proliferation during cancer progression. Proliferation is controlled by cell cycle in normal tissues (40). Numerous regulatory pathways contribute to the cell cycle and their alterations are necessary for cancer cells to overcome the control of the normal cell cycle (Fig. 4). miRNAs may alter the cell cycle by controlling regulators of these regulatory pathways. In this section, the roles of miRNAs in breast cancer cell cycle regulation will be discussed.

**Figure 5:**
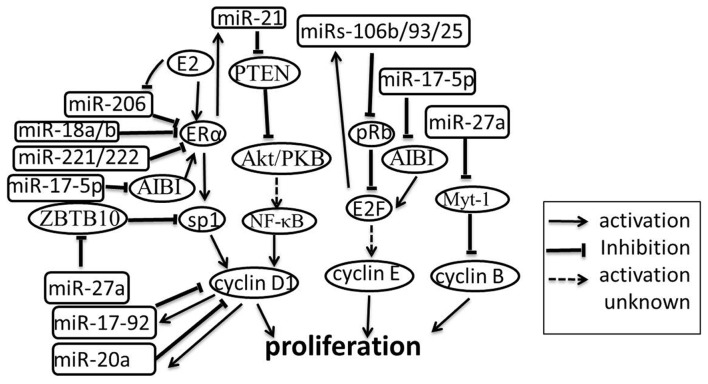
Model for microRNA-involved proliferation in breast cancer.

The cyclin/CDK (cyclin dependent kinase) pathway is an important pathway in the regulation of the cell cycle. This pathway can be regulated by several miRNAs in breast cancer and in cell lines. For example, the miR-17-5p/miR-20a miRNA cluster is shown to attenuate cyclin D1 through directly combining with the 3′-UTR binding site in MCF-7 cell line, thereby inhibiting S-phase entry and halting cell proliferation. Correspondingly, the miR-17/20 cluster is down-regulated and promotes cell proliferation in breast cancer cells (41). Further studies reveal a novel regulatory mechanism in which cyclin D1 induces an miRNA signature including miR-17/20 through the binding of the miR-17/20 promoter region (42). In addition to the miR-17/20 cluster, miR-27a is also associated with the cyclin/CDK pathway. ZBTB10 and Myt-1 are identified as direct targets of miR-27a. ZBTB10 (a putative Sp repressor) can inhibit the proliferation of breast cancer cells by suppressing cyclin D1 indirectly and Myt-1 can block cell cycle progression at the G2/M phase through suppression of cyclin B (43). E2Fs are critical regulators of the cell cycle; they can activate the expression of the miRs-106b/93/25 cluster. E2F is a downstream target of pRb and miRs-106b/93/25 can silence pRb. Furthermore, miR-17-5p is down-regulated in breast cancer cell lines, which has been shown to limit the oncogene AIB1, which enhances the transcriptional activity of the estrogen receptor (ER) and E2F1, leading to proliferation suppression (44). Thus, a negative feedback loop is generated (Fig. 5) (45).

Another cell cycle regulatory pathway in breast cancer is the E2/ERα/Sp1 pathway (46). Upon activation of the receptor estrogen receptor α (ERα), the pathway ERα/Sp1 enhances proliferation via activating cyclin D1, which eventually leads to the G1/S-phase transition. The regulation between the ER and miRNAs has been extensively investigated.

miR-206 is up-regulated in ERα-negative breast tumors and cell lines and inhibits ERα translation by binding to the 3′UTR of ERα mRNA (47). In addition to miR-206, ERα mRNA is also a direct target of miR-18a, miR-18b, miR-193b, miR-302c and miR-221/222 in breast cancer cells. Similar to miR-206, miR-18a, miR-18b and miR-221/222 are also up-regulated in ERα-negative cell lines, suggesting an important role of these miRNAs in the development of ERα-negative breast cancers (48,49). The latter study also suggested that miR-206 expression was strongly inhibited by ERα agonists (E2 and PPT), but not by an ERβ agonist (DPN) and progesterone in MCF-7 cells. This finding suggests the existence of a feedback loop between ER and miRNAs (47). In contrast, E2 increased the expression of miR-21 and Let-7 family members in ERα positive breast cancers (50). A previous publication reported that E2 can down-regulate miR-21 expression and thus increases the protein expression of miR-21 target genes programmed cell death 4 (PDCD4), PTEN and Bcl-2 in MCF-7 breast cancer cells (51). Whatever the reasons for this discrepancy, an attractive speculation is that multiple signaling pathways exist in the regulation of breast cancer cell growth linked to ERs and miRNAs. E2-induced activated ERα directly binds to the miR-21 promoter sequence and increases the levels of miR-21 (50), synchronously recruiting other transcriptional cofactors that bind to target DNA elements thus affecting cell growth in ERα-positive breast tumors. In ERα-negative breast tumors, the E2/ERα signaling pathway is blocked and miR-21 will be decreased. These events may lead to the activition of other ER isoforms which induce the expression of some miRNAs, such as miR-206, miR-18a, miR-18b, miR-221 and miR-222, leading to further inhibition of ERα expression (49)and the activation of other pathways controlling cell growth and proliferation. For the complex regulation of cell proliferation, miRNAs and coregulators are up- or down-regulated by a variety of interacting mechanisms, the investigation of which has only begun. Additional studies need to be undertaken for a more in-depth understanding.

## 4. microRNAs in breast cancer metastasis

**Figure 6:**
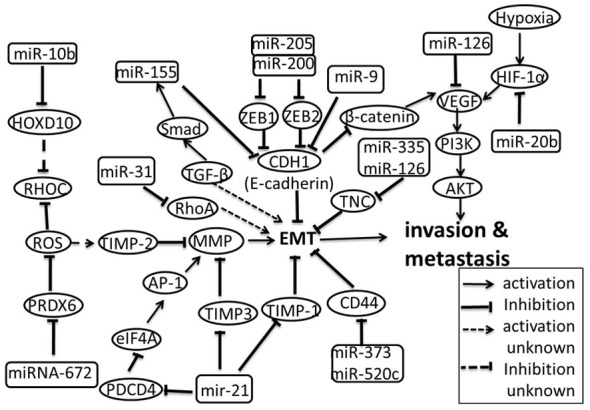
Network for microRNA-involved invasion and metastasis in breast cancer.

After initiation and progression, cancer cells will proceed to the final step: invasion and metastasis (52,53). To initiate the process, tumor cells must first penetrate the epithelial basement membrane and then invade the interstitial stroma. Traversal of basement membranes may require the epithelial-mesenchymal transition (EMT). Distant metastases require tumor-induced angiogenesis that allows for expansion of the primary tumor and permits easy access to the vascular compartment due to defects in the basement membrane of newly formed vessels (54). Many factors, including miRNAs, have been identified as regulators in these processes in different human tumor types. This section will focus on the functions of these factors in breast cancer (Fig. 6).

EMT is characterized by loss of cell adhesion. E-cadherin plays an inhibitory role for cell adhesion molecules (CAM) in metastasis and mediates cell-cell binding. Loss of E-cadherin is a marker that EMT is involved in the progression of carcinoma *in situ* to invasive breast cancer (55,56). miR-9 directly targets CDH1, which is the E-cadherin coding gene, leading to increased cell motility and invasiveness of SUM149 human breast cancer cells (57). Zinc finger E-box binding homeobox 1 (ZEB1) and ZEB2 are shown to be crucial EMT activators in breast cancer by inhibiting E-cadherin expression (58). The miR-200 family and miR-205 directly target ZEB1 and ZEB2, suggesting that down-regulation of these miRNAs is an essential early step in metastasis (59). In addition, a recent study indicates that miR-31 prevents metastasis at multiple steps by inhibiting the expression of prometastatic genes. RhoA is one of such genes, which may enhance EMT in human breast cancer (60). In contrast, miR-155 is overexpressed in breast cancer by the TGF-β/Smad4 pathway and mediates TGF-β-induced EMT by directly targeting RhoA in NMuMG cells. Further study suggests that miR-155 also mediates EMT by indirectly down-regulating E-cadherin (61). These findings reveal that RhoA functions as the target of both tumor suppressor miRNAs (miR-31) and oncogenic miRNAs (miR-155). Both the up- and down-regulation of RhoA contribute to the EMT in different cell lines, suggesting that RhoA regulates EMT in a multiphasic manner.

For invasion to take place, cyclic attachment to matrix components must be released. Metalloproteinases (MMP) play an important role in this event. MMP can degrade the ECM, which is the extracellular part of tissue and mediates cell attachment (62). The tissue inhibitor of metalloproteinases (TIMPs) inhibits the activity of MMP (63) and contains a consensus miR-21 binding site. Previous study reported that miR-21 directly targets TIMP3 in glioma cells and leads to increases of their migratory and invasive abilities (64,65). A recent study showed for the first time that miR-21 negatively regulates TIMP3 expression in breast cancer via the binding of the 3′UTR of TIMP3 mRNA and promotes breast cancer invasion in multiple cell lines *in vitro* (66). In addition, miR-21 also affects invasion and metastasis by directly suppressing expression of tropomyosin 1 (TPM1), PDCD4 and maspin (67). As an actin-binding protein, TPM1 is capable of stabilizing microfilaments and controlling cell motility (68). The actin microfilaments are components of the cytoskeleton, and mediate a variety of essential biological functions in all eukaryotic cells, including providing the driving force for cells (69). TPM1 mRNA expression has been shown to be reduced in the metastatic breast cancer MDA-MB-231 and MDA-MB-435 cell lines and in metastatic colon cancer SW620 cell line (70). These facts suggest that suppression of TPM1 expression by miR-21 is a general way for metastatic tumor cells to disrupt the ECM and contribute to metastasis. Similarly, PDCD4 expression is blocked by miR-21 in breast cancer and colon cancer, suggesting that this interplay may be a general carcinogenic pathway, rather than a tissue-specific mechanism (71,72). The mechanism by which PDCD4 regulates cell invasion remains unclear, however, present evidence supports that PDCD4 inhibits AP-1 by binding to the eukaryotic translation initiation factor 4A (eIF4A). Subsequently, AP-1 and other cis-acting elements together interact with the AP-1 site at the MMP promoter (73,74). Earlier evidence suggests that miR-373 and miR-520c can stimulate migration and invasion of MCF-7 and MDA-MB-435 cells, at least in part through direct suppression of CD44 (53), which functions as a cell surface receptor for several ECM components and mediates cell-cell or cell-substrate interactions through recognition of elements of the ECM (75,76). In addition, miR-335 and miR-126 are also reported to be associated with the ability of breast cancer cells to metastasize to the lung and bone by directly suppressing the ECM component tenascin c (TNC) (77). Taken together, these observations reveal that miRNA can induce cell migration and invasion by directly degrading the ECM or disrupting recognition between the cell and the ECM.

RhoC is an extensive researched prometastatic gene (78,79), which is reported a member of the Ras superfamily of small GTPases, playing a role in modulating assembly of actin-myosin contractile filaments and focal adhesion complex. Ma and colleagues (80) initially observed that miR-10b is highly expressed in metastatic breast cancer cells and they further found that the miR-10b is induced by Twist and it inhibits translation of the mRNA encoding HOXD10 (a homeobox transcription factor that promotes or maintains a differentiated phenotype in epithelial cells). RhoC increases with the decrease of HOXD10 leading to tumor cell invasion and metastasis (80,81). A later study indicated that RhoC is dispensable for tumor initiation but essential for metastasis (78). As a protein with both glutathione peroxidase and phospholipase A2 activities, peroxiredoxin (PRDX) 6 was previously described playing a crucial role in reactive oxygen species (ROS) resistance. Lehtonen *et al* (82) first demonstrated that PRDX may be associated with human lung carcinoma. Chang and colleagues (83) found that PRDX6 is up-regulated in highly invasive and potentially metastatic MDA-MB-231 HM breast cancer cells compared with their parental cells. Furthermore, they demonstrated that overexpression of PRDX6 in breast cancer cells promoted their invasive and metastatic potential *in vitro* and *in vivo* (82,84). RhoC was up-regulated and TIMP-2 was down-regulated, in the cells with up-regulation of PRDX6 (83). When PRDX6 was knockdown by miRNA-672, RhoC was down-regulated and TIMP-2 was up-regulated (83). These findings indicate that miRNA-672 indirectly regulates breast cancer cell invasiveness and metastasis via down-regulating PRDX6 expression. However, how PRDX6 regulates RhoC and TIMP-2 as well as whether PRDX6 is an instigator of metastasis or merely a correlative product during progression of breast cancer are still beyond present understanding (85). As the available knowledge has shown that PRDX6 functions as an anti-oxidative protein to protect cells from damage by ROS, therefore it is reasonable to believe that ROS may play a key role in the regulation of RhoC and TIMP-2 by PRDX6. Further study into the mechanism of these relationships may add to the current state of knowledge of these signaling pathways, and will improve our understanding of metastasis-related interaction between miRNAs and cancer protein-coding genes.

To obtain sufficient nutrients and oxygen for metastasis of solid tumors, the formation of new blood vessels (angiogenesis) is necessary (86). It is now well established that tumor-induced angiogenesis is driven by the overexpression of angiogenic factors such as vascular endothelial growth factor (VEGF), which is the most potent inducer of angiogenesis. Of their wide range of biological actions, the role of miRNAs in tumor angiogenesis has received the greatest attention. Recent studies have shown that VEGF-A may be well-regulated by miR-126 in normal tissues and miR-126 was restrictly expressed in human breast cancer where the VEGF/PI3K/AKT signaling pathway was vigorously activated. In addition, miR-126 directly targeted VEGF and its expression was decreased in human breast cancer, revealing that miR-126 plays a role in angiogenesis (87–90). Yet, another pathway regulating VEGF expression was presented by Ma *et al* (57) who described that the up-regulation of VEGF-A mRNA by miR-9 depends on its ability to down-regulate E-cadherin expression and to activate β-catenin-mediated transcription. E-cadherin has been identified as the direct target of miR-9 and VEGF-A has been described as a transcriptional target gene of β-catenin. The data illustrates a novel mechanism by which miR-9 promotes angiogenesis through stimulation of VEGF-A expression in breast cancer. A recent study proposed that the VEGF expression in breast cancer cells is mediated by HIF-1 in a miR-20b-dependent manner. Hypoxia is one of the features within the tumor microenvironment. Hypoxia inducible factor1 (HIF-1) is a heterodimeric transcription factor consisting of HIF-1α and HIF-1β subunits (91,92). Under oxygenated conditions, HIF-1α is rapidly degraded, while in hypoxic conditions, this factor is stabilized and contributes to angiogenesis by directly activating the VEGF gene (93). Taking into account the above discussed evidence on the involvement of miRNAs in breast cancer-induced angiogenesis it would be of interest to address the functional relationship among these miRNAs. These regulators adjust the same target-VEGF by different pathways under specific conditions and trigger angiogenesis. One interesting question to be addressed is whether these miRNAs share the same specific expression pattern or not. Therefore, more novel miRNAs which participate in the process of VEGF-mediated angiogenesis in breast cancer should be identified to understand these expression patterns.

## 5. Conclusion

Breast cancer develops because of complex multistep processes. Generally speaking, there are three phases (initiation, progression and metastasis) in the complex multistep process. These phases are composed of a sequence of events, including self-renewal apoptosis, cell cycle and mobility. miRNAs are an evolutionarily conserved class of small, approximately 22-nucleotide non-coding RNAs that decrease gene expression post-transcriptionally in a sequence-specific manner, which participates in these events and some members extensively contribute to breast tumorigenesis. Over the past years, the utilizations of high-throughput technologies, such as microarray, a large number of ectopic miRNAs have been observed in breast cancer but the critical roles of most of these miRNAs remain largely unknown. Among these miRNAs, one miRNA can potentially regulate the expression of hundreds of genes, and on the other hand, a single transcript can be targeted by multiple miRNAs. However, knowledge of how an miRNA simultaneously down-regulates multiple proteins in the same pathway and an understanding of the miRNA target genes and their biologic functions is limited. There is no doubt that to further understand breast cancer pathogenesis, identifying the genome-wide targets of these miRNAs is essential. In addition, identifying the factors determining tissue-and cell-specific expression of miRNAs is also pivotal. As described above, miRNAs may act cooperatively through multiple target sites in one gene (94), or one miRNA may regulate a group of functionally related genes. Interestingly, unwanted cross-reaction does not appear in these two regulatory patterns. In addition, the mechanism does not seem to be specific of miRNA itself but the specific expression of miRNA. If only partially complementary sequences exist, targets will be repressed, regardless of the target gene specificities. In fact, miRNAs are expressed in specific cells and tissues at specific developmental stages and conditions. These facts clearly show that the system determining the specific expression of miRNAs plays a key role in regulating gene expression. In recent years, numerous miRNAs and their targets have been confirmed in breast cancer and have been recognized as new therapeutic targets. However, these new therapeutic targets may not work, for the destruction of interplay between one miRNA and its target will be restored by another miRNA with similar function. Here, we postulate that the miRNA specificity determining system is composed of the effective targets, and miRNAs function as signal molecules together with other regulatory elements mediating breast cancer tumorigenesis in a stage-specific manner. Nevertheless, at the current stage little is known about these systems and various aspects of them need to be clarified in a future study. Taken together, the miRNA specificity determining system may serve as more effective potential target for breast cancer therapy in comparison with miRNA.

## Figures and Tables

**Table I:** microRNAs and their targets in different phases.

Phase	microRNA	Expression and role	Target	Refs.
Initiation	miR-200c	Down-regulated, BT-IC self-renewal suppressor	Bmi-1	(20)
	Let-7	Down-regulated, BT-IC self-renewal suppressor	Ras	(21)
	miR-30	Down-regulated, BT-IC self-renewal suppressor	Ubc9, integrin β3	(22,23)
Progression	miR-21	Up-regulated, anti-apoptotic factor	Unknown	(33)
	miR-145	Down-regulated, inducing apoptosis	RTKN	(35)
	miR-155	Up-regulated, anti-apoptotic factor	FOXO3a	(36)
	miR-34a	Down-regulated, inducing apoptosis	Bcl-2	(39)
	miR-17/20	Down-regulated, proliferation suppressor	Cyclin D1	(41,42)
	miR-27a	Up-regulated, inducing proliferation	Myt-1, ZBTB10	(43)
	miR-17-5p	Down-regulated, proliferation suppressor	AIBI	(44)
	miRs-106b/93/25	Up-regulated, inducing proliferation	pRb	(45)
	miR-206	Up-regulated, inducing proliferation	ERα	(47)
	miR-18a/b/221/222	Up-regulated, inducing proliferation	ERα	(48,49)
	miR-21	Up-regulated, inducing proliferation	PTEN	(50)
Metastasis	miR-205/200	Down-regulated, EMT suppressor	ZEB1, ZEB2	(59)
	miR-31	Down-regulated, EMT suppressor	RhoA	(60)
	miR-155	Up-regulated, inducing EMT	E-cadherin	(61)
	miR-21	Up-regulated, inducing EMT	TIMP1, TIMP3 PDCD4	(66,67)
	miR-373/miR-520c	Up-regulated, inducing EMT	CD44	(53)
	miR-335/miR-126	Up-regulated, inducing EMT	TNC	(77)
	miR-10b	Up-regulated, inducing EMT	HOXD10	(80)
	miRNA-672	Down-regulated, EMT suppressor	PRDX6	(83)
	miR-126	Down-regulated, angiogenesis suppressor	VEGF	(87)
	miR-9	Up-regulated, inducing EMT and angiogenesis	E-cadherin	(57)
	miR-20b	Down-regulated, angiogenesis suppressor	HIF-1α	(92)

BT-IC, breast cancer-initiating cells; EMT, epithelial-mesenchymal transition.
